# Partial response after treatment with Conversion chemotherapy: A case report of a patient with colon cancer and unresectable hepatic metastases

**DOI:** 10.1097/MD.0000000000015239

**Published:** 2019-04-26

**Authors:** Zhaoting Bu, Cheng Lu, Xu Yang, Hao Lai, Yuting Jiang, Yicheng Li, Yuan Lin, Yuzhou Qin, Minxi Xiao, Chunfeng Cheng, Qi Liu

**Affiliations:** a Affiliated Tumor Hospital of Guangxi Medical University; b Department of Gastrointestinal Surgery and Guangxi Clinical Research Center for Colorectal Cancer, Affiliated Tumor Hospital of Guangxi Medical University, Nanning, Guangxi Autonomous Region, China.

**Keywords:** case report, colorectal cancer, hepatic metastasis, partial response

## Abstract

**Rationale:**

Many studies have reported radical resection for liver metastasis and the primary tumor could represent an important prognostic factor in patients affected by colorectal liver metastases (CRLM). However, resection of huge liver metastases from colon cancer has been seldom reported.

**Patient Concerns:**

A 58-year-old man presented with huge liver metastases from colon cancer. Laboratory tests revealed elevated tumor markers and a wild-type mutation in the K-RAS gene. A computed tomography scan demonstrated unresectable liver masses with a 16.5-cm maximum diameter and intrahepatic duct dilatation due to compression by the liver metastases.

**Diagnosis:**

The patient was diagnosed with stage IV descending colon carcinoma with multiple huge hepatic metastases.

**Interventions:**

He was administered 3 treatment courses, including 9 cycles of combined chemotherapy with mFOLFOX6 plus cetuximab (mFOLFOX6 + Cet), and the liver masses reduced. After a preoperative assessment by a multidisciplinary team when the 9 cycles of systemic chemotherapy had been completed, the patient underwent hepatectomy, followed 4 months later by a laparoscopic colectomy. We used a reverse strategy (liver-first) for the patient.

**Outcomes:**

In this case, liver-first treatment (systemic chemotherapy of mFOLFOX6 + Cet) was an effective treatment for unresectable CRLM. No postoperative complications occurred. The patient continued to receive postoperative chemotherapy (mFOLFOX6 + Cet) at the latest follow-up. During the 17 months of follow-up, tumor recurrence was un-noted.

**Lessons:**

Treating colorectal cancer patients with huge hepatic metastases is possible, and surgeons should consider various treatment options in the management of these patients.

## 1 Introduction

Colorectal cancer (CRC) is the third leading cause of cancer death in the world.^[1,2]^ Approximately, 25% of patients with CRC also present with CRC liver metastases (CRLM) at the time of first diagnosis, and up to 50% further develop recurrence in the liver during the disease course.^[3]^ Liver metastasis is the leading cause of death in these patients, whose overall 5-year survival is only 5%.^[4]^ Resection of the metastasis is the only treatment that offers the possibility of cure, and has been proven to contribute to patient survival. However, only 10% to 15% of the initial CRLM are considered resectable. In the remaining cases, the current trend is to downstage initially unresectable metastases by systemic chemotherapy.^[3,5]^

To date, few studies have shown that preoperative chemotherapy can effectively shrink tumors, aiming to prolong overall survival after systemic chemotherapy.^[5–7]^ Here, we report a case of a patient who had descending colon carcinoma with multiple huge hepatic metastases and was treated with a combination of FOLFOX6 plus cetuximab using a liver-first approach.

## 2 Methods

### 2.1 Patient information

The study was approved by the Ethics Committee of The Affiliated Tumor Hospital of Guangxi Medical University. A 58-year-old man presented with dull pain in the lower abdominal region and hematochezia. His medical record indicated a 6-year history of hypertension treated with regular medication; his family history was unremarkable. Written informed consent was given by patient.

### 2.2 Clinical findings and diagnostic assessment

**Figure 1:**
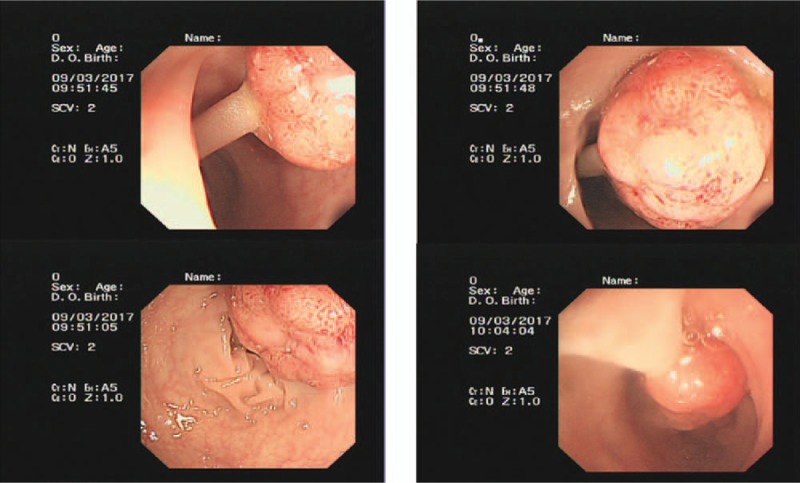
Fiber colonoscopy showed an advanced cancer in the descending colon, blockage of the intestinal cavity, and multiple colonic polyps.

**Figure 2:**
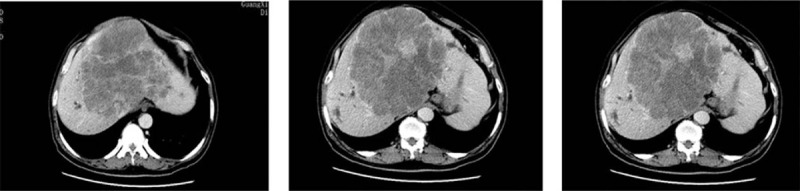
Computed tomography showed multiple hepatic metastases and a future liver remnant <30%.

Laboratory studies showed an elevated blood count (white blood cell 12.49 × 10^9^/L, platelet 442 × 10^9^/L) and elevated tumor markers carcinoembryonic antigen (CEA) 21.9 ng/mL; his Child–Pugh stage was A. Gene detection showed a wild-type mutation in the K-RAS gene. Fiber colonoscopy showed an advanced cancer in the descending colon, blockage of the intestinal cavity, and multiple colon polyps (Fig. 1). Biopsy revealed a siphonate adenocarcinoma. Computed tomography showed multiple hepatic metastases 16.5 × 15.6 cm in size, intrahepatic duct dilatation caused by compression by the liver metastases, and a future liver remnant <30% (Fig. 2). Pathological findings were moderately differentiated adenocarcinoma in the splenic flexure, and a tubular adenoma and tubulovillous adenoma distant from the tumor at 30 cm and 10 cm, respectively, from the anal verge. Thus, the patient was diagnosed with stage IV descending colon carcinoma with multiple huge hepatic metastases.

### 2.3 Therapeutic intervention

**Figure 3:**
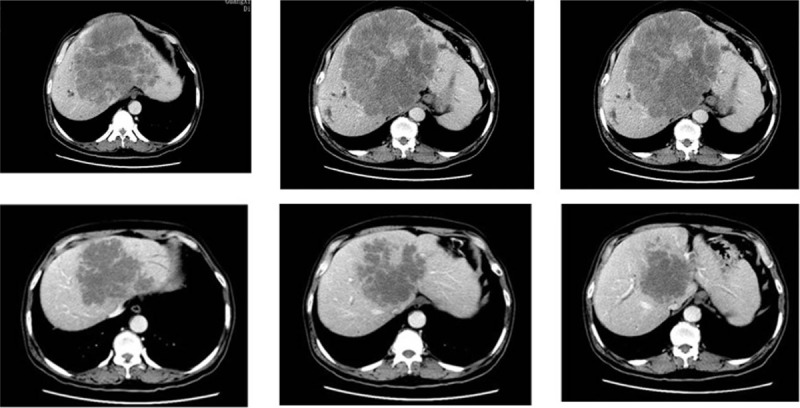
Three cycles of chemotherapy (mFOLFOX6 +Cet) resulted in partial response of the huge CRLM to treatment. CRLM = colorectal liver metastases.

Treatment effect was evaluated by the Response Evaluation Criteria in Solid Tumors (RECIST) guidelines,^[8]^ including progressive disease, stable disease, partial response, and complete response. Treatment consisting of a combination of 3 cycles of chemotherapy and target drugs (mFOLFOX+Cet) resulted in partial response of the huge CRLM to treatment (Fig. 3). Tumor markers, including CEA, declined to normal levels. Hepatobiliary surgery specialists of the multidisciplinary team (MDT) considered the future liver remnant to be insufficient and recommended continuation of the chemotherapy, with the aim of converting the initial unresectable CRLM to a resectable CRLM in order to improve the R0 resection odds of this patient.

**Figure 4:**
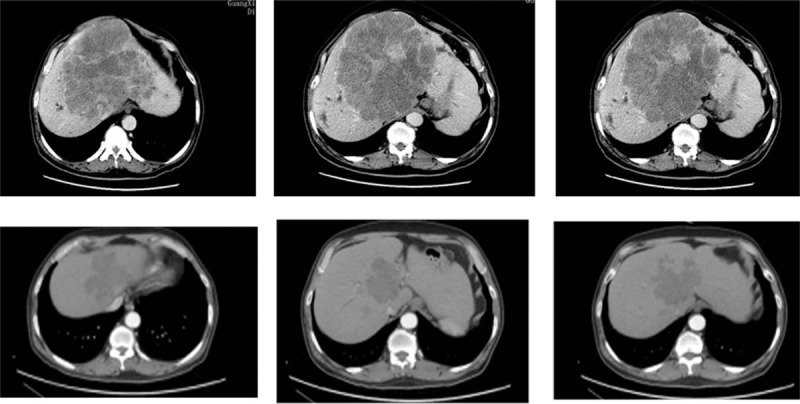
After 6 cycles of mFOLFOX6+Cet, the CRLM continued to show partial response to treatment. CRLM = colorectal liver metastases.

**Figure 5:**
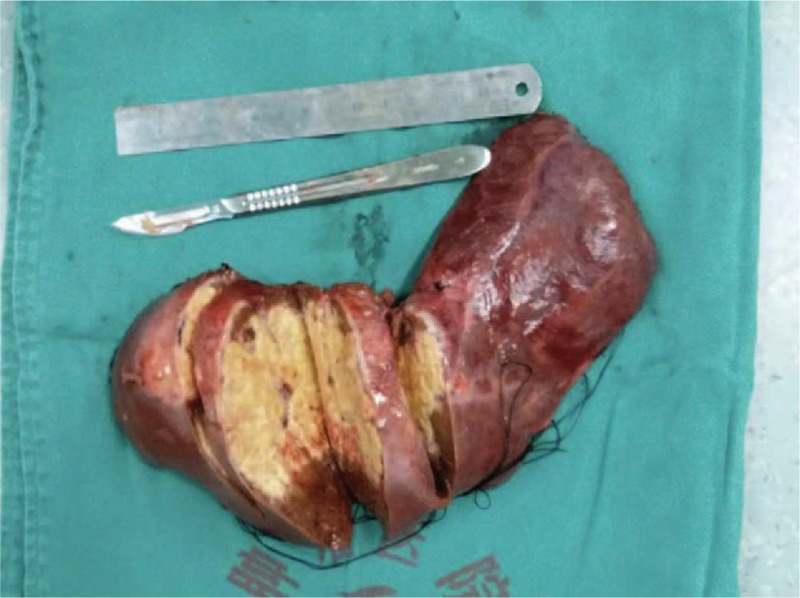
The patient underwent hepatectomy, which yielded resected hepatic tissue 15 cm in length.

After 6 cycles of chemotherapy (mFOLFOX6+Cet), the CRLM continued to show a partial response (decrease of ≥30% in tumor size) to treatment (Fig. 4), so the patient underwent hepatectomy (Fig. 5).

Chemotherapy toxicity was assessed at each cycle using the Common Terminology Criteria for Adverse Events (CTCAE, v5.0),^[9]^ which is divided into 5 grades, grade III and above being considered severe. During the chemotherapy, only grade I was noted (leukopenia) and no severe adverse events occurred.

### 2.4 Follow-up and outcomes

During the postoperative period, no severe postoperative complications, including liver complications, were noted. Following the partial tumor response observed during chemotherapy, he underwent descending hemicolectomy in January 2018.

From May 2017 to October 2018, the duration of follow-up after the left hemicolectomy was 22 months. The patient was alive and well at the final follow-up. Further systemic therapy was administered at the latest follow-up. To date, no recurrence has been documented and the patient was satisfied with the treatment effect.

## 3 Discussion

Surgical resection of CRLM offers patients the best likelihood of cure, with one case study reporting an average 5-year survival rate of 47% after resection,^[10]^ and only 15% to 35% of patients having indications for hepatectomy.^[4]^

The NCCN clinical practice guidelines in oncology for colon and rectal cancers recommend FOLFOX as the optimal treatment for patients with unresectable recurrent colon cancer and hepatic metastasis. A combination of systemic chemotherapy with FOLFOX and anti-VEGF antibody or anti-EGFR antibody allows the rescue of 12.5% of patients with unresectable CRLM via hepatectomy.^[3,11]^ Neoadjuvant chemotherapy for initially unresectable CRLM can also improve the hepatectomy rate.^[12,13]^ Consistent with previous studies,^[6,14,15]^ our patient showed partial response to chemotherapy (mFOLFOX6+Cet), and radical surgery was performed for the primary tumor and liver metastases.

Liver-first surgery may be particularly applicable to CRC patients with synchronous liver metastases, where preoperative long-course chemoradiotherapy for the colorectal primary prior to surgical resection creates a potential ‘window’ in which liver resection can be undertaken.^[13,16]^ The liver-first strategy may also be oncologically advantageous as it addresses the hepatic metastatic burden before progression in the liver leads to unresectability. Another potentially important benefit of the liver-first approach is that pelvic surgery may be either avoided or be less extensive in patients with rectal tumors, with a complete endoscopic, radiological, and clinical response to chemoradiotherapy.^[17]^

In this case, after completing 6 cycles of chemotherapy, with reduction in the CRC liver mass, the choice of an appropriate time to resect the liver metastasis tumor remained unclear. An MDT, including specialists in gastrointestinal surgery and hepatobiliary surgery and members from the departments of intervention, imaging diagnosis, radiotherapy, pathology, and oncology, discussed the best treatment option for the CRLM. The liver metastasis was close to the vena cava according to computed tomography, so continued chemotherapy was considered an appropriate strategy.

Our case suggests an equal effectiveness of combined systemic chemotherapy and the reverse strategy following MDT consultation in clinical practice. The present case is unique and had a favorable prognosis, suggesting a more appropriate treatment direction for patients with huge/multiple CRLM.

However, a limitation of the present case report is the absence of long-term follow-up. A 3-year or 5-year follow-up duration could have more adequately confirmed the treatment's curative effect.

Treating CRC patients with huge hepatic metastases is possible, and surgeons should consider various treatment options in the management of these patients.

## Author contributions

**Conceptualization:** Yuan Lin and Yuzhou Qin, Zhaoting Bu.

**Data curation:** Cheng Lu, Xu Yang.

**Formal analysis:** Zhaoting Bu, Yuting Jiang.

**Investigation:** Zhaoting Bu, Yicheng Li.

**Methodology:** Zhaoting Bu, Yicheng Li, Yuan Lin.

**Supervision:** Zhaoting Bu, Hao Lai.

**Visualization:** Minxi Xiao, Chunfeng Cheng, Qi Liu.

**Writing – original draft:** Zhaoting Bu, Cheng Lu and Xu Yang

**Writing – review editing:** Zhaoting Bu, Hao Lai and Yuzhou Qin
